# Growth of phylogenetically diverse microalgae under Far-Red light enriched spectra: implication for space missions’ sustainability

**DOI:** 10.3389/fmicb.2026.1833797

**Published:** 2026-05-19

**Authors:** Beatrice Boccia, Mariano Battistuzzi, Elisabetta Liistro, Riccardo Claudi, Lorenzo Cocola, Luca Poletto, Nicoletta La Rocca

**Affiliations:** 1 Department of Biology, University of Padua, Padua, Italy; 2 National Institute for Astrophysics, Astrophysical Observatory of Arcetri (INAF-OAA), Florence, Italy; 3 National Institute for Astrophysics, Astronomical Observatory of Padua (INAF-OAPd), Padua, Italy; 4 National Research Council – Institute for Photonics and Nanotechnologies (CNR–IFN), Padua, Italy

**Keywords:** acclimation, BLSS, Far-Red, light spectra, pigments, red-dwarfs, space sustainability

## Abstract

**State of the art:**

Photosynthetic oxygenic microorganisms are recognized to be the functional core of Bioregenerative life support systems (BLSS) for sustaining long-term human presence in space. Among these organisms, microalgae are particularly promising due to their elevated growth rates, highly efficient light use, and ability to synthesize a plethora of valuable compounds. Their cultivation could lead to the sustainable production of food enriched in essential nutraceuticals while ensuring oxygen regeneration in confined environments. Understanding their responses to different light spectra is crucial for optimizing their cultivation in close systems and reducing the energetic costs. Considering the growing interest in the potential contribution of Far-Red (FR) light to the photosynthetic process and biomass production, we investigated the acclimations of different microalgal species under light spectra including this low-energy waveband in combination with visible (VIS) light.

**Research methodologies:**

Four phylogenetically diverse microalgal species, either capable (*Chromera velia* and *Nannochloropsis gaditana*) or incapable (*Dixoniella grisea* and *Chlorella vulgaris*) of utilizing solely FR light beside VIS radiation, were cultivated for 10 days under three light regimes: (i) simulated solar light (SOL); (ii) low-VIS, FR-enriched light (FR-e); (iii) FR light peaking at 730 nm (FR). All treatments were characterized by the same total photon flux, but differed in spectral distribution and total available energy. The culture growth during acclimation to different lights was assessed. At the beginning and at the end of experiments, samples were investigated through optical microscopy, spectrophotometry and HPLC techniques. Changes in cell morphology and photosynthetic apparatus *in vivo* absorption of cultures were analyzed. Photosynthetic pigments were characterized and quantified.

**Results:**

The results showed that microalgal growth under FR-e conditions was consistently higher than expected, being not proportional to the reduced fraction of available VIS photons (37.0% in FR-e vs. 84.3% in SOL). Specifically, growth under FR-e with respect to SOL, reached 61% in *C. vulgaris*, 82% in *D. grisea*, 91% in *N. gaditana*, and 93% in *C. velia*. Considering that the total energy of the FR-e spectrum is 83.5% of SOL (expressed in W m^−2^) this light regime results particularly advantageous for the cultivation of FR-users microalgal species. The assessment of cell features and pigment composition showed similar characteristics of the produced biomass in SOL and FR-e for all tested species. Finally, the experiments also revealed a FR dose dependent photoacclimation in *C. velia*, whose FR light absorption capacity was enhanced at increasing FR doses.

**Key findings:**

The results demonstrate that the growth of diverse microalgal species is differently influenced by a low-VIS FR-enriched spectrum. For FR-user species under this light regime the energy costs can be reduced for the efficient production of biomass, while maintaining a comparable quality. The most promising species turned out to be *N. gaditana*, due to its high growth rate and the capability to use FR light beside the VIS one. These findings are crucial for a sustainable microalgal cultivation in the frame of BLSS.

## 1 Introduction

It is generally recognized that the in loco provisioning for future Moon and Mars missions could be ensured by establishing self-sustaining ecosystems within circular bioregenerative life support systems (BLSS) (Skoog, 1984; Verseux et al., 2022). In this context, the oxygenic photosynthetic microorganisms, including cyanobacteria and microalgae, serve a dual key function by supporting the production of food and high-value biomolecules while simultaneously contributing to recover and maintain air quality through the fixation of carbon dioxide and the release of molecular oxygen. The combination of their capacity for biomass production and gas regulation, together with their minimal growth requirements, constitutes a fundamental principle in the development of space biotechnology applications (Fahrion et al., 2021).

These microorganisms represent an advantageous option compared to plants, exhibiting faster growth rates under equivalent light energy, water and resources availability. Although cyanobacteria are often preferred for their resilience to extreme environmental conditions (Yadav et al., 2022), their genetic engineering can still present some limitations in the molecular toolbox currently available (Cheng et al., 2023). In contrast, microalgae, as eukaryotic organisms, display a broader array of metabolic pathways for the biosynthesis of valuable metabolites and show a high potential for genetic engineering and long-term stability (Ayub et al., 2025). Several microalgal species are already employed on Earth to produce biomass enriched in essential nutraceutical compounds, such as proteins, carbohydrates, lipids and pigments with antioxidant activity (Ayub et al., 2025). Numerous ground-based experiments have already demonstrated that diverse microalgae can be successfully integrated into self-sustaining ecosystems for space missions, efficiently recovering nutrients and other essential minerals from astronauts’ biological waste and from lunar and Martian soil simulants (Casula et al., 2024; Fais et al., 2022; Tao et al., 2022). However, besides nutrient supply, both the intensity and spectral quality of light represent crucial requirements that deserve careful consideration in the cultivation of photosynthetic microorganisms. In fact, the photon use efficiency varies among species and strongly affects biomass productivity and energy costs. Far-Red (FR) light, in particular, is energetically advantageous compared to visible (VIS) light, as it requires lower energy input for generation, making it use an economically favorable option for large-scale cultivation. Recent evidence demonstrated that combining VIS and FR photons in cultivation lights can enhance biomass productivity in plants (Huber et al., 2024; Li et al., 2021). Positive results have been shown also for cyanobacteria. *Chlorogloeopsis fritschii* exhibited a twofold increase in biomass accumulation when FR light was supplemented to white light (WL) (Silkina et al., 2019). Beneficial effects on growth have been reported also for *C. fritschii*, *Scynechocystis* sp. PCC 6803 and *Synechococcus* sp. PCC 7335 when exposed under high FR light combined with very low VIS light (Battistuzzi et al., 2023a; Liistro et al., 2024). On the other hand, only a limited number of studies have been carried out so far to investigate the growth responses of microalgae under these kinds of spectra. In *Chlorella vulgaris*, the addition of FR light at 740 nm to blue and red wavelengths significantly enhanced biomass productivity (Kula et al., 2014). Increased growth rates and accumulation of carotenoids were recorded for *Dunaliella bardawil* cultures exposed to WL combined with FR light with respect to solely WL, under the same irradiance (Sánchez-Saavedra et al., 1996).

These findings delineate an emerging research frontier to which our study contributes, aiming at understanding whether the integration of different levels of VIS and FR light can effectively affect microalgal growth and whether the use of specific spectral combinations may provide tangible advantages in BLSS systems. Insights on algal responses to FR-enriched spectra also find applications in the astrobiological question on the possibility of oxygenic photosynthesis to function outside the Solar System. In fact, most of the potentially habitable Earth-like exoplanets have been discovered orbiting red-dwarf stars, which emit light spectra predominantly composed of FR photons, with a minor VIS component (Shields et al., 2016). Consequently, any hypothetical life on their exoplanets’ surfaces would likely be exposed to this type of irradiance, which brings to the question of whether oxygenic photosynthesis could be sustained on these planetary bodies. Additionally, within our Solar System, the search for extinct or extant life on Mars targets its subsurface environments, sheltered from harsh surface conditions (Viúdez-Moreiras, 2021), where FR photons penetrate more effectively than VIS light, providing filtered irradiance suitable for photosynthetic activity (Behrendt et al., 2020; Gan and Bryant, 2015; Ohkubo and Miyashita, 2017).

In this framework, we previously performed preliminary screening tests in solid medium that showed a positive effect of high FR light combined with very low VIS light on the growth of some microalgae (Battistuzzi et al., 2023b).

In the present work we deepened our investigations by assessing the growth rates, pigment content and photoacclimation strategies of four microalgal species (*Chlorella vulgaris*, *Dixoniella grisea*, *Nannochloropsis gaditana* and *Chromera velia*), cultured in liquid medium under light spectra differing in both total irradiance and photon wavelength composition. The three illumination conditions utilized were: (i) a simulated solar spectrum (SOL), characterized by a high proportion of photons in the VIS range and low level of FR light, representing the most energetic condition; (ii) a mixed spectrum, named Far-Red enriched (FR-e), in which a reduced fraction of VIS photons was combined with high levels of FR radiation and (iii) a FR spectrum peaking at 730 nm with the lowest total energy, usable for oxygenic photosynthesis only by a few number of species.

The chosen FR-e spectrum accurately reproduces both the light emitted by a red-dwarf star type M7 (Battistuzzi et al., 2023a), and the light conditions experienced by organisms in naturally shaded environments where the propagation of shorter wavelength is limited due to absorption by the superficial layers. Such types of environments include microbial mats, forest understories, subsurface niches, and dense photobioreactor cultures (Gan and Bryant, 2015; Ohkubo and Miyashita, 2017; Saccardo et al., 2022). In all these cases, the VIS light is strongly attenuated, while FR photons dominate.

In this study we tested species from distinct phylogenetic lineages that are either capable or incapable of utilizing FR light to drive the photosynthetic process.

*Dixoniella grisea* and *Chlorella vulgaris,* derived from a primary endosymbiosis, cannot perform FR-driven photosynthesis.

*Dixoniella grisea* was selected as a representative of Rhodophyta, distinguished for its singular pigment profile, which includes, besides chlorophyll and carotenoids, phycobiliproteins (PBPs), in particular phycoerythrin and phycocyanin. The antioxidant, photoprotective and bioactive properties of PBPs make them valuable compounds for nutraceutical, cosmetic, and biomedical applications (Tounsi et al., 2023). Beyond its pigment profile, *D. grisea* produces highly viscous extracellular polymeric substances (EPS), rich in polysaccharides and proteins. They have been identified as a potential source of eco-friendly bio-additives to be used in lubricant formulations (Gavalás-Olea et al., 2021). EPS can also be used as biocementing agents of granular materials, such as regolith. On Earth they are applied in bioconstruction to enhance the stabilization of soil particles (Khoshtinat, 2026).

*Chlorella vulgaris,* on the other hand, is widely used in biotechnology, due to its high growth rate and versatile metabolic profile, to produce high-value compounds. In particular, its biomass is characterized by high protein content, accounting for approximately 40–58% of dry weight, but also contains a wide array of vitamins and antioxidant compounds, enhancing its nutraceutical properties (Fais et al., 2025; Safi et al., 2014; Wang et al., 2024). Its potential in space-oriented biotechnologies has already been tested in multiple spaceflight missions and ground-based studies (Belz et al., 2014; Cycil et al., 2021; Helisch et al., 2020; Niederwieser et al., 2018; Windisch et al., 2026), in which its suitability for long-term cultivation in photobioreactors was evaluated (Fahrion et al., 2021).

The responses of these two non-FR-user species were compared in this study with two species known to exploit FR light, *Chromera velia* and *Nannochloropsis gaditana*.

*Chromera velia* represents the microalgal lineages of the Chromerida, strictly correlated to Apicomplexa, not previously utilized in biotechnology and space research and still relatively understudied compared with well-characterized biotechnological models like *N. gaditana and C. vulgaris*. It is one of the few microalgal species so far demonstrated capable of driving oxygenic photosynthesis under FR light. As a photosynthetic symbiont of scleractinian corals, *C. velia* naturally experiences such light conditions, inhabiting light-filtered microenvironments within host tissues, where the spectral distribution is shifted toward longer wavelengths (Wangpraseurt et al., 2012). Its ability to live free or persist within the coral holobiont could make it a candidate for future studies on carbon biomineralization. Moreover, recent research has shown that *C. velia* can cope with high CO₂ levels, exhibiting increased biomass production and improved nutrient use efficiency, without altering the relative abundance of major organic pools in the cell (Venuleo et al., 2018). These traits may be relevant for space biotechnology applications involving carbon recycling and low energy availability.

Finally, *Nannochloropsis gaditana*, has been widely investigated for biotechnological purposes due to its remarkable capacity to synthesize human-relevant molecules including omega-3 fatty acids with anti-inflammatory and nutritional properties or accumulate neutral lipids, often exceeding 50% of its biomass, under optimized conditions such as nitrogen starvation. These compounds are valuable for both nutritional and industrial applications, among which the production of biofuel. *N. gaditana*, however, has never been considered as a biological support for space missions despite its widespread utilization in terrestrial biotechnologies. Nevertheless, most recent studies have proven that this species is also able to photosynthesize relying only on monochromatic FR light (Liistro et al., 2026), potentially expanding the spectral range to support its growth if cultivated in VIS-FR combined spectra and thus also increasing its relevance in biotechnological strategies for space sustainability.

To verify the capacity of each selected strain to sustain culture proliferation and characterize the biomass produced under these different light regimes and energy levels available for photosynthesis, we assessed growth performances, analyzed cell phenotype, recorded *in vivo* absorption spectra to evaluate changes in photosynthetic apparatus light-harvesting properties, and performed spectrophotometric and HPLC analyses to evidence any possible shift in pigment composition associated with photoacclimation.

Beyond advancing our understanding of the physiological limits or advantages of photosynthesis under alternative spectral conditions, these results provide new insights for space missions sustainability, identifying microalgal species capable of sustaining growth and maintaining photosynthetic biomass quality at reduced energy costs.

## 2 Materials and methods

### 2.1 Microalgal strains and maintenance cultivation conditions

The research has been carried out using the following 4 microalgal strains: *Dixoniella grisea* SAG 72.90, *Chlorella vulgaris* CCAP211-11B, *Nannochloropsis gaditana* CCAP849/5, and *Chromera velia* CCAP1602/1.

All organisms were maintained in liquid medium in a climate-controlled chamber. *C. vulgaris,* a freshwater microalga, was grown in BG-11 medium (Rippka et al., 1979), whereas the other marine species (*D. grisea, N. gaditana,* and *C. velia*) were cultured in F/2 medium (Guillard and Ryther, 1962). Guillard’s Marine Water Enrichment Solution 50X (G0154, Sigma-Aldrich, Darmstadt, Germany) was added to the F/2 medium with a final concentration of 1X. The cultures were maintained at about 28–30 °C under continuous illumination provided by a white-fluorescent lamp (OSRAM W840, Premstaetten, Austria) with a light intensity of 30 μmol photons m^−2^ s^−1^ in the 380–780 nm range. Cultures were renewed once a week to maintain the exponential growth phase.

Both temperature and light intensity were set within the optimal range for the growth of all the species tested (Bamba et al., 2015; Eggert et al., 2007; Levasseur et al., 2023; Lukeš et al., 2017; Meng et al., 2021; Rocha et al., 2003).

### 2.2 Experimental plan

**Figure 1:**
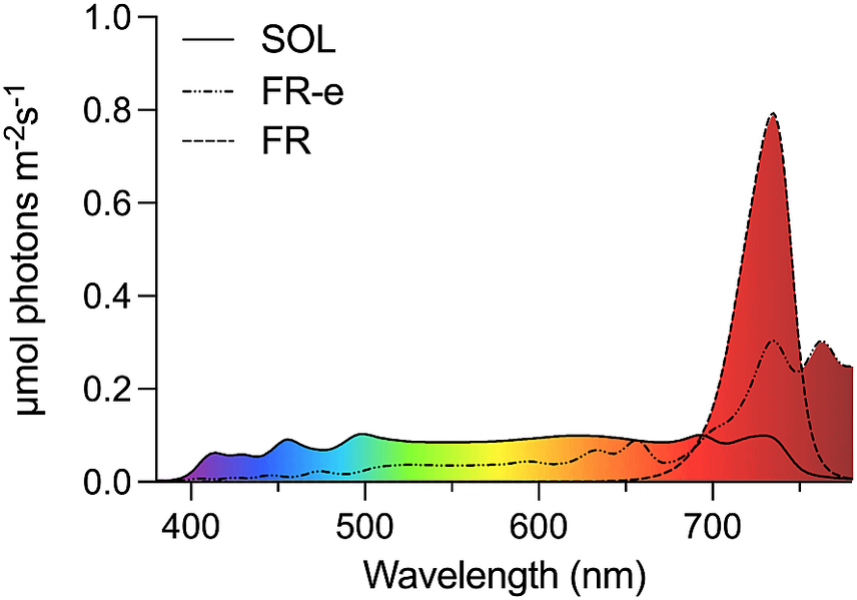
Emission spectra of SOL light (continuous line), FR-e light (dash-dot line) and FR light spectrum peaked at 730 nm (dashed line) emitted by the simulators and registered with the LI-COR 180 spectrometer (LICOR) in the range 380–780 nm.

The maintenance cultures of each strain were first acclimated for a minimum of 7 days to SOL simulated light spectrum and then utilized to prepare the *inocula* for at least 3 biological replicates for each light condition. At the starting day of the experiment (T0) the cells of *C. vulgaris*, *D. grisea*, *C. velia* and *N. gaditana* were inoculated in flasks of 250 mL containing 50 mL of culture in the respective fresh specific growth medium to obtain an optical density (OD) of 0.3 at 750 nm and exposed for 10 days under the three light conditions at 28–30 °C. The control culture was maintained under the SOL light while the treatment cultures were transferred to less energetic light spectra (FR-e or FR) with different photon distributions (Figure 1). Light was provided in continuous at the intensity of 30 μmol photons m^−2^ s^−1^, in the 380–780 nm range for all the tested spectra.

Growth was monitored by measuring the OD at 750 nm (OD_750_) of each microalgae culture throughout the 10-day experimental period, for all light conditions.

For each replicate the biomass was collected at the initial (T0) and final (T10) time points for subsequent analyses, including cell features observed at light and fluorescence microscope, *in vivo* absorption spectra, pigment (chlorophylls, carotenoids, and, for *D. grisea,* phycobiliproteins) quantification and characterization at the spectrophotometer and HPLC.

### 2.3 Light sources

Three different custom-built devices were utilized to provide the light spectra tested. FR-e light spectrum was generated using the previously detailed Star Light Simulator (Battistuzzi et al., 2020; Claudi et al., 2020), while the simulators of the SOL spectrum and the monochromatic FR light were described in (Battistuzzi et al., 2023a).

The SOL light spectrum covered the range between 365 and 750 nm, the FR-e one the 350–850 nm range, while the FR one the 365–800 nm, to include all the wavelength potentially relevant for the photosynthesis (Figure 1). Light intensity was set to provide an equivalent photosynthetic photon flux density (PPFD) of approximately 30 μmol photons m^−2^ s^−1^ measured through a spectrometer (LI-COR 180, LI-COR) (Ecosearch Srl, Italy) in the range of 380–780 nm.

With respect to common illumination setups, like white fluorescent or LED lamps, characterized by emission spectra with unnaturally pronounced peaks at certain wavelengths, the employed SOL simulation system includes the FR band and generates a continuous distribution of photons, ensuring a flux below the saturation threshold of photosynthesis for all species at the light intensity set.

The three spectra differed substantially in the quality and total energy of the incident radiation. The SOL spectrum was characterized by 84.8% of photons (25.3 μmol photons m^−2^ s^−1^) within the VIS range (400–700 nm), corresponding to the higher-energy fraction absorbed by photosynthetic pigments, while the remaining photons (15.2%) were distributed in the FR region (700–780 nm). The FR-e spectrum had a marked shift toward longer wavelengths, with 37.2% of photons (11.1 μmol photons m^−2^ s^−1^) within the VIS region and the majority of photons (18.8 μmol photons m^−2^ s^−1^) emitted at low-energy in the FR region. The spectrum providing the lowest energy was generated by the FR lamp peaking at 730 nm, which included only 7.6% of photons (2.3 μmol photons m^−2^ s^−1^) within the 400–700 nm range, the spectral region absorbed by most photosynthetic pigments (Supplementary Table 1).

The total energy (in W m^−2^) calculated for each light spectrum was: 6.3 W m^−2^ SOL, 5.26 W m^−2^ FR-e, and 4.83 W m^−2^ FR (Supplementary Table 1).

The simulated SOL and FR-e spectra provided a realistic and well-distributed photon flux across the VIS and FR ranges that organisms can experience in natural environments.

### 2.4 Growth assessment

To assess the growth of the cultures, OD was measured at five time points (days 0, 2, 4, 7, and 10). OD was determined on appropriately diluted samples, to avoid measurement saturation, using a double-beam spectrophotometer (Cary 100 UV–Vis, Agilent, Santa Clara, CA, USA) calibrated against the culture medium as a blank. Absorbance was measured at 750 nm to minimize the interference of photosynthetic pigments. Each measurement was performed in at least 3 biological replicates.

The average growth rate ( μ ) was calculated as follows:

$$\text{Average}\;\;\;\text{growth rate}\;\mu =\frac{\log_{e\;\;}(n_{f})-\log_{e\;\;}(n_{i})}{t_{f}-t_{i}}$$

Where n_i_ and n_f_ indicate the OD at the initial and final day of growth. t_i_ and t_f_ correspond to the days these measurements were taken.

The maximum growth rate was calculated as the maximum value obtained for each timepoint of the growth curve, calculated with the following equation:

$$\text{Maximal growth rate μmax}=\frac{\log_{e\;\;}(n_{2})-\log_{e\;\;}(n_{1})}{t_{2}-t_{1}}$$

Where n_2_ and n_1_ indicate the OD measured at consecutive time points t_1_ and t_2_ within the exponential growth phase.

### 2.5 Cell feature assays

To assess cell features, an optical microscope (DMI4000, Leica Microsystems, Wetzlar, Germany) was used, equipped with a Leica DFC7000T camera. Images were obtained at 100x magnification in brightfield and fluorescence (autofluorescence of chlorophyll *a* was detected in the red spectral range). Samples were prepared at T0 and upon the end of the experiment by fixing 1 mL of culture with 20 μL of buffered formaldehyde, they were then stored in the dark at 4 °C until analysis.

The diameter of cells was determined as follows. For *N. gaditana* and *D. grisea*, with a regular cell size, automated measurements of diameters were performed with a Cellometer Auto X4 cell counter (Nexcelom Bioscience, Lawrence, MA, USA) in a 4 μL culture sample. For *C. vulgaris* and *C. velia*, which present, respectively, a high variability of shapes and marked aggregation of cells, the diameters were measured manually (at least 15 cells) using FIJI-IMAGEJ (National Institutes of Health), through the ‘straight’ IMAGEJ tool in the brightfield images. Additional manual measurements, to confirm the data obtained by the Cellometer were performed also for *N. gaditana* and *D. grisea* with the same procedure.

### 2.6 *In vivo* spectroscopy

*In vivo* absorption spectra of cultures were recorded at day 0 (T0) and at the end of each experiment (T10). For each algal species, 3 mL of culture were centrifuged at 3500 × g for 10 min at 24 °C. The resulting pellet was gently homogenized and resuspended in 600 μL of the appropriate culture medium (BG-11 or F/2) to concentrate the culture at desired OD values and eliminate cell debris. Spectra were acquired between 350 and 750 nm using an Agilent Cary 100 UV–VIS spectrophotometer (Agilent, Santa Clara, CA, USA) calibrated with the corresponding culture medium as a blank. Spectra were recorded with the optical glass cuvettes oriented with the opaque (opaline) side facing the probing ray to correct for light scattering, as described by Amesz et al. (1961), Gan et al. (2014), Liistro et al. (2026), and Shibata (1959).

### 2.7 Pigments extraction and quantification

For the quantification of total carotenoids and chlorophylls, 5 mL of culture were centrifuged at 10,000 × *g* for 10 min at 24 °C. The resulting pellet was washed twice with Milli-Q water to remove any residual culture medium salts and then resuspended in 1 mL of dimethylformamide (DMF) to extract lipophilic pigments. Samples were incubated overnight at 4 °C in the dark to prevent pigment photodegradation.

For the extraction of the phycobiliproteins in *D. grisea*, 5 mL of culture were centrifuged at 17,500 × *g* for 10 min at 4 °C. The resulting pellet was mixed with an equal volume of glass beads (acid washed, 150–212 μm, Sigma, Darmstadt, Germany) in 1X extraction buffer (0.01 M Na₂HPO₄, 0.15 M NaCl). Cell disruption was performed using a Bead-Beater (BioSpec Products, Bartlesville, OK, USA) for three cycles of 10 s at maximum oscillation speed (3,500 OPM), with 30 s incubation on ice between rupture cycles. After the addition of 150 μL of phosphate buffer, a final cell lysis step was performed with an additional 4 s Bead-Beater cycle.

Lysed samples were centrifuged at 17,500 × *g* for 10 min at 4 °C to separate the pellet from the supernatant. After supernatant recovery, 200 μL of phosphate buffer were added to the pellet, vortexed, and centrifuged again. This procedure was repeated until the supernatant appeared transparent, indicating complete pigment extraction.

Absorption spectra of extracts were recorded between 350 and 750 nm using an Agilent Cary 100 UV–VIS spectrophotometer (Agilent, Santa Clara, CA, USA). Appropriately diluted samples were measured in optical glass cuvettes after baseline correction with the extraction buffer.

Pigment concentrations in *Chlorella vulgaris*, which contains chlorophyll *b*, were calculated using the following equations (Porra et al., 1989; Wellburn, 1994):

$$\mathrm{Chl}\;a\;[\frac{\mathrm{\mu g}}{\mathrm{ml}}]=12\times A664-3.11\times A647$$

$$\mathrm{Chl}\;b\;[\frac{\mathrm{\mu g}}{\mathrm{ml}}]=20.78\times A647-4.88\times A664$$

$$\mathrm{Ca}r_{\mathrm{tot}}\;[\frac{\mathrm{\mu g}}{\mathrm{ml}}]=\frac{\left(1000\times A480-1.12\times \mathrm{Chl}\;a-34.07\times \mathrm{Chl}\;b\right)}{245}$$

Where Chl *a*, Chl *b* and Car_tot_ indicate, respectively, Chlorophyll *a*, Chlorophyll *b*, and total carotenoids.

For *D. grisea, N. gaditana,* and *C. velia*, which contain only chlorophyll *a*, chlorophyll and carotenoids quantification was performed using the equations described (Chamovitz et al., 1993; Porra et al., 1989; Wellburn, 1994):

$$\mathrm{Chl}\;a\;[\frac{\mathrm{\mu g}}{\mathrm{ml}}]=11.92\times A664$$

$$\mathrm{Ca}r_{\mathrm{tot}}\;[\frac{\mathrm{\mu g}}{\mathrm{ml}}]=A461-(0.046\times A664)\times 4$$

Phycobiliprotein (PBP) concentrations in *D. grisea* were calculated using the following equations (Bennett and Bogorad, 1973).

$$\mathrm{PC}\;[\frac{\mathrm{\mu g}}{\mathrm{ml}}]=\frac{\left(A615-0.474\times A652\right)}{5.34}$$

$$\mathrm{APC}\;[\frac{\mathrm{\mu g}}{\mathrm{ml}}]=\frac{\left(A652-0.208\times A615\right)}{5.09}$$

$$\mathrm{PE}\;[\frac{\mathrm{\mu g}}{\mathrm{ml}}]=\frac{\left(A562-2.41\times \mathrm{PC}-0.849\times \mathrm{APC}\right)}{9.62}$$

Where PC, APC and PE indicate, respectively, Phycocyanin, Allophycocyanin and Phycoerythrin.

Final pigment concentrations were normalized to the sampled culture volume and corrected for dilution factors, when applied.

All the procedures were carried out under low ambient light to prevent pigment degradation.

### 2.8 Reverse phase high -performance liquid chromatography (RP-HPLC)

For RP-HPLC analysis, 5 mL of sample were centrifuged at 17,500 g for 10 min at 4 °C. After discarding the supernatant, the pellet was washed twice with Milli-Q to remove any residual culture medium salts. The pellet was then mixed with an equal volume of glass beads (150–212 μm, Sigma) using 90% acetone. It underwent 5 cycles in a Bead-Beater (BioSpec Products, Bartlesville, OK, USA) for 20 s each at maximum oscillation speed (3,500 OPM), alternated with 2 min on ice. These steps are designed to disrupt the cells and extract hydrophobic pigments compatible with the buffer. An additional 150 μL of 90% acetone was added, followed by a final cell lysis cycle with 20 s in the Bead-Beater. The lysed samples were centrifuged at 17,500 g for 10 min at 4 °C to separate the pellet from the supernatant. After collecting the supernatant, 200 μL of 90% acetone were added to the pellet, vortexed, and centrifuged again. These steps were repeated until the supernatant appeared clear, indicating complete pigment extraction. The extracts were stored at −20 °C in the dark until analysis.

Analyses were conducted with an Agilent 1,100 series LC with a column (250 mm length, 4 mm diameter) containing 5 μm silica particles coated by C-18 atom chains (Lichrospher 100 RP, Merck) as the stationary phase. The elution of the pigments of *C. vulgaris*, *D. grisea* and *N. gaditana* was obtained using the mobile phase consisting of solvent A (86.8% acetonitrile 9.6% methanol, 3.6% Tris–HCl 0.1 M, pH8) and solvent B (80% methanol, 20% hexane) with a gradient from solvent A to solvent B run from 9 to 12.5 min at a flow rate of 2 mL min (Färber and Jahns, 1998). For *C. velia* a longer protocol was utilized, for the correct separation of the pigments. The mobile phase was constituted by two solutions: buffer A (42% methanol, 33% acetonitrile, 25% HPLC grade H_2_O) and buffer B (50% methanol, 20% acetonitrile and 30% ethyl acetate in ratio 50:20:30). The two solutions were eluted in the column according to the protocol reported in (Gan et al., 2014). Chromatograms were registered at 440 nm. The DAD detector enabled the registration of the absorption spectrum of each eluant, and their identification was based on literature (Alboresi et al., 2016; Basso et al., 2014; Jeffrey et al., 1997; Moore et al., 2008; Quigg et al., 2012; Serive et al., 2017; Takacs et al., 2025; Wright et al., 1991).

### 2.9 Statistical analyses

Statistical analyses were performed through the software GraphPad Prism v.10.1.0 (GraphPad). Means and standard deviations were calculated for at least 3 different biological replicates per light condition (SOL, FR-e and FR lights). Comparison between the light conditions were carried out using the Ordinary One-Way ANOVA analyses followed by Tukey’s multiple comparisons test. When small sample sizes (*n* = 3–4) and unequal variances occurred and multiple comparisons were less reliable, an unpaired *t*-test with Welch’s correction was used for specific comparisons between each dataset. Welch’s ANOVA followed by the Games–Howell *post hoc* test was used to confirm pairwise comparisons.

## 3 Results

### 3.1 Culture growth and phenotype assessment

**Figure 2:**
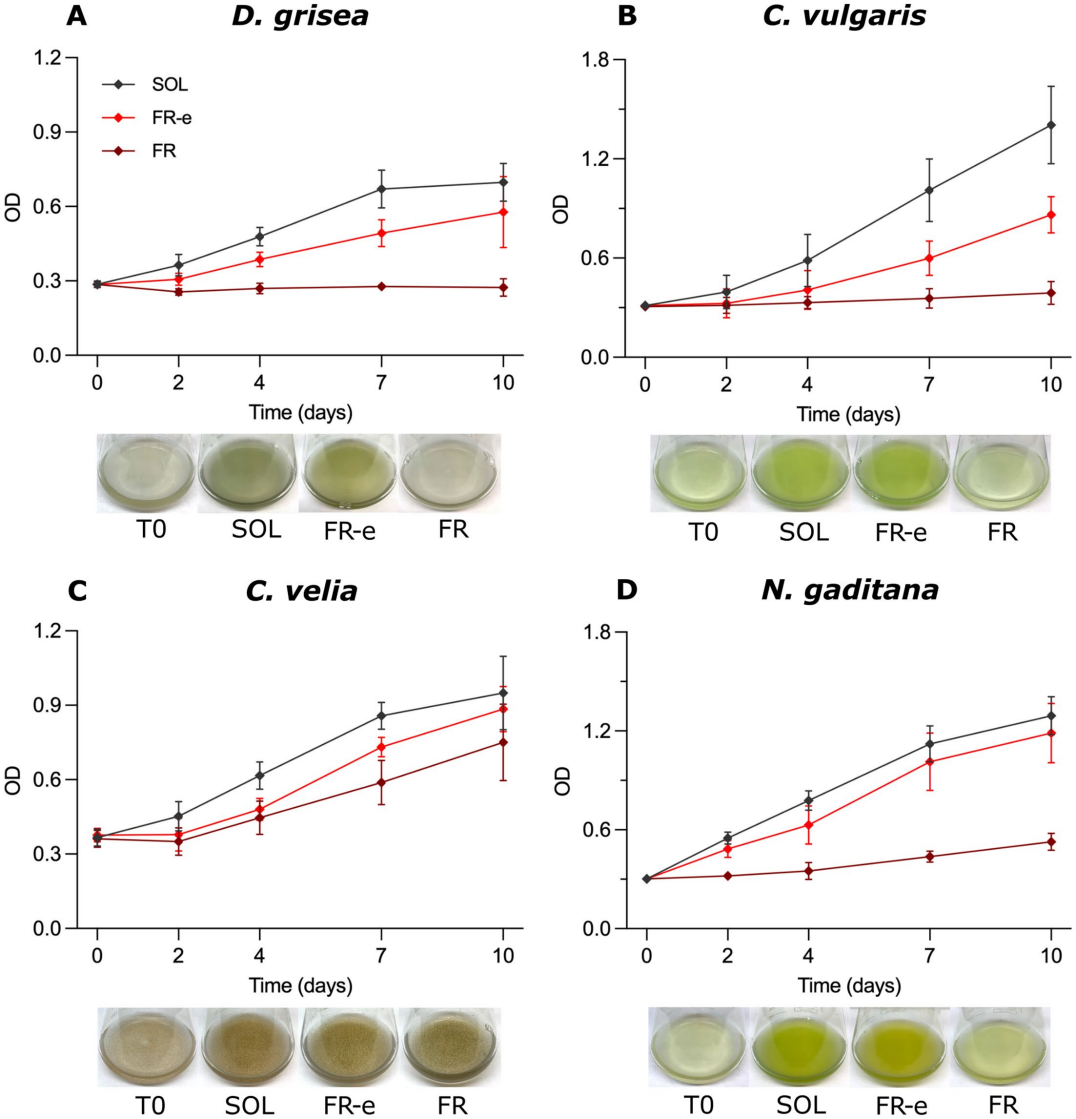
Growth curves of *D. grisea* **(A)**, *C. vulgaris* **(B)**, *C. velia* **(C)** and *N. gaditana* **(D)** obtained measuring the optical density (OD) at 750 nm during the acclimation to SOL, FR-e and FR light spectra. Data are presented as the mean with standard deviation of the OD_750_ measurements of at least 3 biological replicates. Below each growth curve, the corresponding representative images of culture phenotypes at the inoculum (T0) and after 10 days (T10) of exposure to each light are shown.

Microalgal growth responses varied under the different light spectra tested (Figure 2). Higher differences in growth among the four microalgal species were observed under FR light. After 10 days of exposure (T10), both *D. grisea* and *C. vulgaris* showed negligible OD changes under FR, the first one maintaining a stable OD (0.3 ± 0.03) until the end of the experiment and the second one reaching a final OD mean value of 0.39 ± 0.07. In *C. velia* and *N. gaditana,* instead, a clear culture growth was observed, reaching the OD values of 0.75 ± 0.15 and 0.53 ± 0.05, respectively. Notably, *C. velia* showed growth comparable to that observed under FR-e and SOL spectra.

Phenotypic observations of liquid cultures were consistent with growth curves (Figure 2): in FR conditions *D. grisea* pigmentation did not increase with respect to T0, whereas the color of *N. gaditana* and *C. velia* intensified proportionally to OD. An exception was observed for *C. vulgaris* under this light on the final day of the experiment: although OD increased from 0.3 up to 0.39 ± 0.07, cultures appeared pale, with no evident pigment intensification over time.

On the other hand, under FR-e light, at T10 the OD had increased in all tested strains, independently of their capability to use solely FR light: for *D. grisea*, *C. velia* and *N. gaditana* the OD almost reached the values registered in SOL light, despite the smaller VIS photon fraction which characterizes the FR-e spectrum. After 10 days of exposure to FR-e light, in *D. grisea* the OD value was 0.58 ± 0.14, while, in SOL light, the culture reached OD 0.71 ± 0.08. *C. velia* grew up to an OD of 0.88 ± 0.09 in FR-e and in SOL simulated light to 0.95 ± 0.15. Finally, also for *N. gaditana* OD values under FR-e and SOL were notably similar (respectively 1.18 ± 0.18 and 1.29 ± 0.11).

Differently from the other microalgae, *C. vulgaris* growth under FR-e was substantially reduced with respect to SOL, with OD values of 0.86 ± 0.11 and 1.40 ± 0.23, respectively. Consistently, both under FR-e and SOL light, visual inspection of the cultures confirmed that the pigmentation intensity of all strain liquid cultures was increased at the end of the experiment (Figure 2).

**Figure 3:**
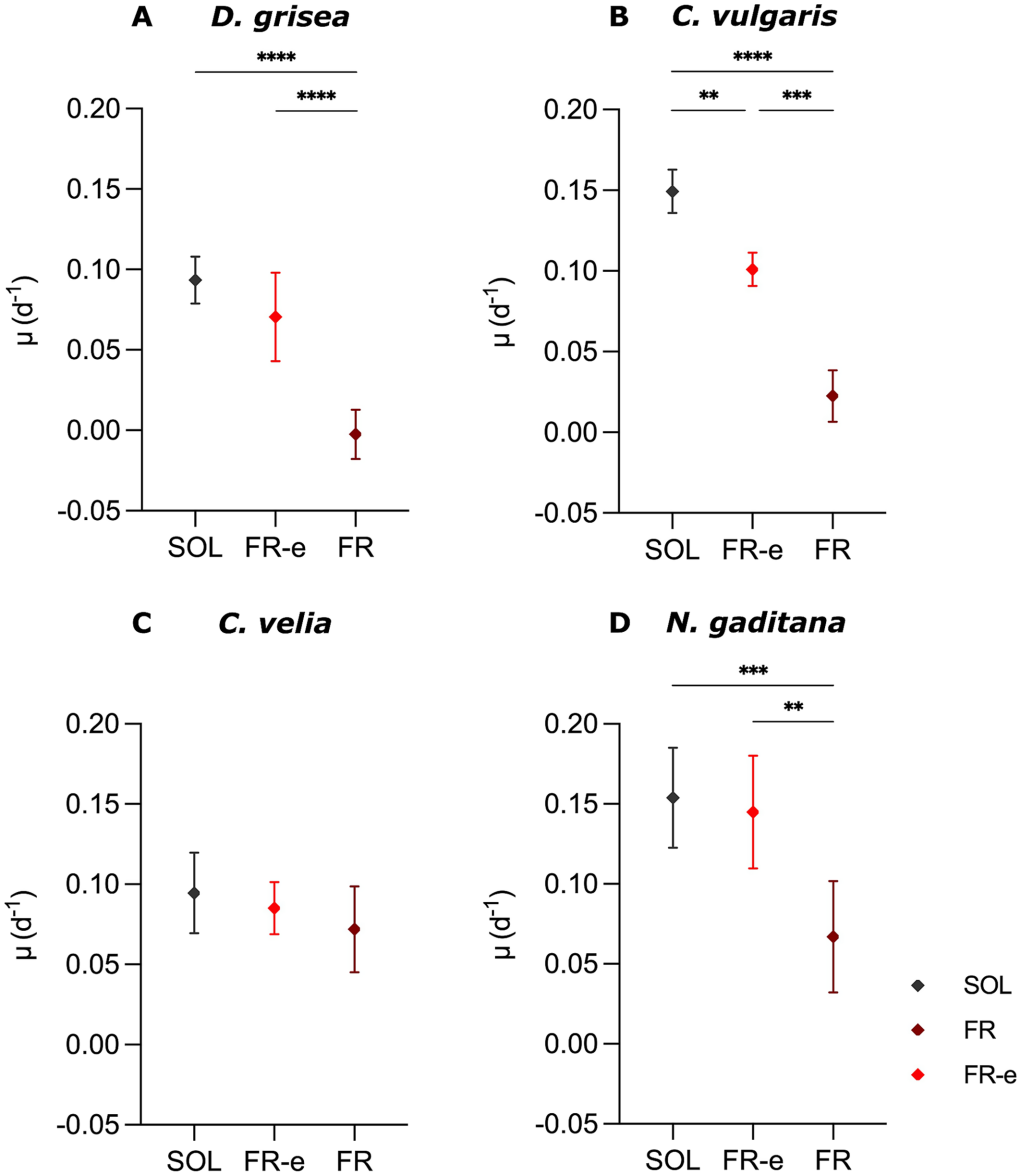
Average growth rates *μ* (d^−1^) of *D. grisea* **(A)**, *C. vulgaris* **(B)**, *C. velia* **(C)** and *N. gaditana* **(D)** cultures after 10 days of exposure (T10) to SOL, FR-e and FR light spectra. Data are expressed as mean and standard deviation of at least 3 biological replicates. Statistical analysis: Ordinary One-Way ANOVA followed by Tukey’s HSD *post-hoc* test (multiple comparisons) or Welch’s ANOVA followed by the Games–Howell *post hoc* test depending on the dataset. Significance levels: **, *p* < 0.01; ***, *p* < 0.001; ****, *p* < 0.0001. Non-significant differences are not shown.

Both the average and maximum growth rates, *μ* and μ_max_ (d^−1^), were calculated starting from OD values, allowing to evaluate overall growth across the entire experiment, as well as the growth performances during the faster exponential phases, respectively. The average *μ* (Figure 3) highlighted that under FR light the growth capacity was significantly lower than under FR-e and SOL spectra in *C. vulgaris* and *N. gaditana*. The lowest growth rate value in FR monochromatic light was registered in *C. vulgaris* with *μ* = 0.02 ± 0.02 d^−1^, while *N. gaditana* showed a more positive response of growth under FR, μ = 0.07 ± 0.03 d^−1^, according to the increase in OD reached at the end of the experiment. *D. grisea*, μ = −0.01 ± 0.01 d^−1^ did not show any growth capacity in this light regime.

*Chlorella vulgaris*, together with *N. gaditana*, had the highest *μ* values in SOL light (respectively 0.15 ± 0.01 d^−1^ and 0.15 ± 0.03 d^−1^), demonstrating a rapid and consistent biomass increase under this spectrum. As observed in the growth curves (Figure 2B) the microalga *C. vulgaris* displayed the most differential growth under all tested light conditions: significant differences were observed between *μ* values in SOL and FR-e, where μ was 0.10 ± 0.01 d^−1^ (Figure 3B). Interestingly, in *N. gaditana*, the average growth rate in FR-e did not change much from that in SOL, being 0.145 ± 0.035 d^−1^.

The findings for *C. velia* differed markedly from the other species. Cells showed a general lower growth capacity if compared with the other strains, but maintaining comparable *μ* values under all the tested conditions, ranging from 0.7 to 0.9 d^−1^.

Analyses of maximum growth rates provided further insights into the interpretation of growth potential for the different microalgae (Supplementary Table 2). During the exponential phase, μ_max_ values for SOL and FR-e did not differ significantly in all the tested species (*p*-value < 0.05), consistent with the average μ values, except for *N. gaditana*. In *C. vulgaris* μ_max_ was also comparable under the two conditions, even if their average μ values calculated over the full experimental period differed significantly (Figure 3B).

**Figure 4:**
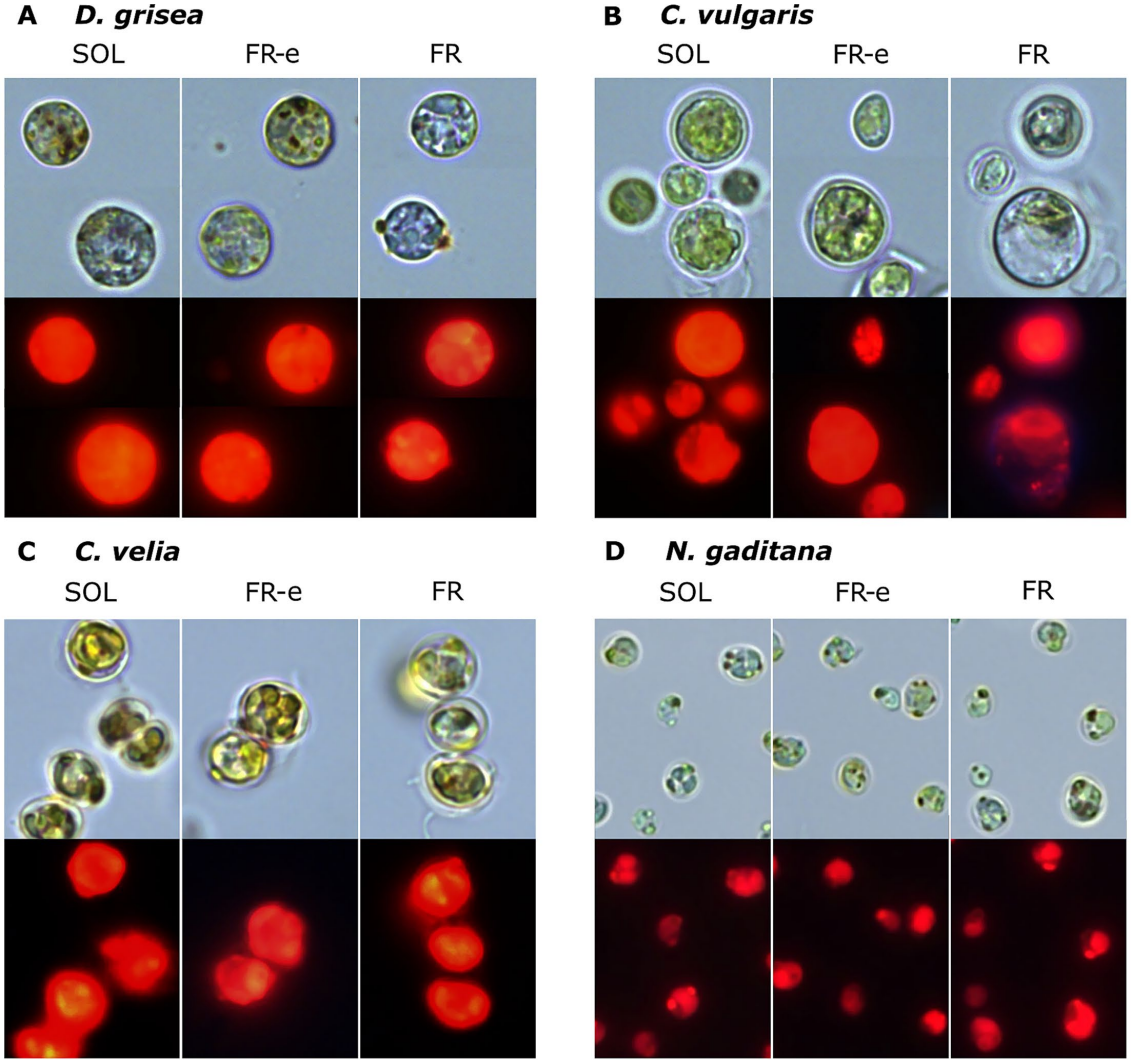
Representative optical microscopy micrographs of *D. grisea*, *C. vulgaris*, *C. velia* and *N. gaditana* of cultures after 10 days of exposure to the different light spectra (respectively SOL, FR-e and FR). Acquisitions in brightfield and in autofluorescence of Chl *a* are shown. Scale bar = 10 μm.

The observation of micrographs and the measurement of cell diameters allowed us to assess possible changes in cell’s morphology and in the chlorophyll autofluorescence levels of microalgae acclimated to the various light spectra (Figure 4).

According to literature, typical cell features were observed in *C. vulgaris* (Safi et al., 2014), *D. grisea* (Yoon et al., 2010), *N. gaditana* (Abdelkarim et al., 2025) and *C. velia* (Moore et al., 2008). Overall, the data obtained measuring diameters indicate that cell size did not vary significantly under the different illumination conditions (Table 1).

**Table 1:** Average and standard deviation of diameters measurements in cells of *D. grisea*, *C. vulgaris*, *C. velia* and *N. gaditana* after 10 days of exposure to SOL, FR-e and FR light spectra.

Species	SOL	FR-e	FR
*D. grisea*	7.86 ± 1.11^a^	8.09 ± 1.11^a^	6.36 ± 0.75^a^
*C. vulgaris*	4.40 ± 1.76^a^	4.15 ± 2.12^a^	3.77 ± 1.80^a^
*C. velia*	4.11 ± 0.70^a^	4.00 ± 0.61^a^	3.80 ± 0.52^a^
*N. gaditana*	3.25 ± 0.87^a^	3.17 ± 0.74^a^	3.40 ± 0.53^a^

Data is expressed in μm, as mean and standard deviation of at least 15 manual measurements for each species and 2 automated ones obtained at the Cellometer for *D. grisea*, and *N. gaditana*. Different letters indicate significant differences among treatments (*p* < 0.05). Statistical analysis: Ordinary One-Way ANOVA followed by Tukey’s HSD *post-hoc* test (multiple comparisons) or Welch’s ANOVA followed by the Games–Howell *post-hoc* test depending on the dataset.

Cell pigmentation observed at the optical microscope appeared similar across all tested conditions for all microalgal species, except for *C. vulgaris* grown under FR light, which showed paler or empty cells. These observations were confirmed by fluorescence microscopy (Figure 4). The images indicated that all microalgal samples retained strong chlorophyll autofluorescence, suggesting good viability of all species under the different illumination conditions. The only exception was *C. vulgaris* grown under FR light, which showed a partial loss of chlorophyll autofluorescence, consistent with the faded colour of the liquid cultures shown in Figure 2B.

### 3.2 Photosynthetic apparatus assessment and pigment composition analyses

**Figure 5:**
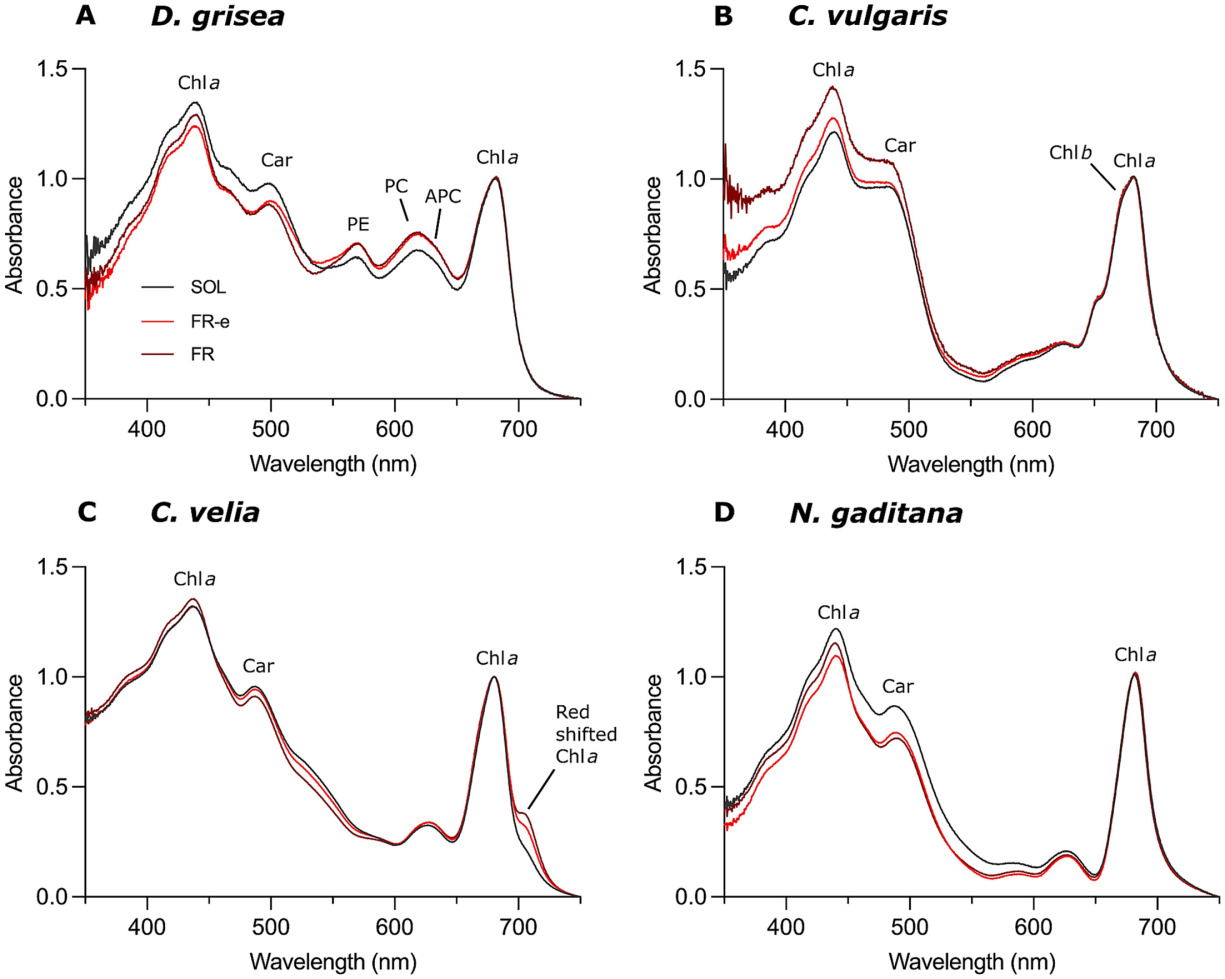
*In vivo* absorption spectra of cultures of *D. grisea* **(A)**, *C. vulgaris* **(B)**, *C. velia* **(C)**, and *N. gaditana* **(D)** at the same OD and collected after 10 days of exposure to SOL, FR-e, and FR light spectra. Chl *a*, Chlorophyll *a*; Chl *b*, Chlorophyll *b*; Car, Carotenoids; PE, Phycoeritrin; PC, Phycocyanin; APC, Allophycocyanin. Spectra are normalized maximum peaks of Chlorophyll *a* in the red (680 nm).

*In vivo* absorption spectra were recorded at the same OD for all microalgal cultures collected at T10 and were normalized to the chlorophyll *a* red absorption peak (Figure 5). Comparative analysis revealed specific photoacclimation responses in each tested strain after exposure to the various light spectra, involving changes in the pigment composition and in the ability of the cells to absorb different light wavelengths. This approach was particularly useful to unravel the photoacclimation to FR light and to different photon fluxes within this waveband of the tested light regimes. To this end, we focused on the *in vivo* absorption capacity of cell cultures at wavelengths >700 nm. No changes were detected in this spectral region for any strain except for *C. velia*. In this species, the rise of an absorption shoulder above 700 nm was observed, which was more pronounced under FR light but also detectable under FR-e treatment.

Analysis of the remaining spectral regions further revealed variations in the relative contribution of different pigments to light absorption. *N. gaditana* and *D. grisea* exhibited enhanced absorption in the carotenoid-associated light-harvesting band (400–550 nm) when grown under SOL with respect to FR-e and FR lights. In contrast, this response was displayed in *C. vulgaris* under FR light. Moreover, *D. grisea* also showed slight changes in the phycobiliprotein absorption region (500–660 nm), with reduced levels under SOL with respect to FR-e and FR spectra.

In all the tested species, chlorophyll and carotenoid concentrations per mL (Supplementary Figure 1) increased as expected, almost reflecting the culture growth (Figure 2). The exceptions (PBPs in *D. grisea*, all pigments in *C. velia* and Chl *a* in *N. gaditana*) are better interpreted with the normalization of pigment content per OD and the pigment ratios.

**Figure 6:**
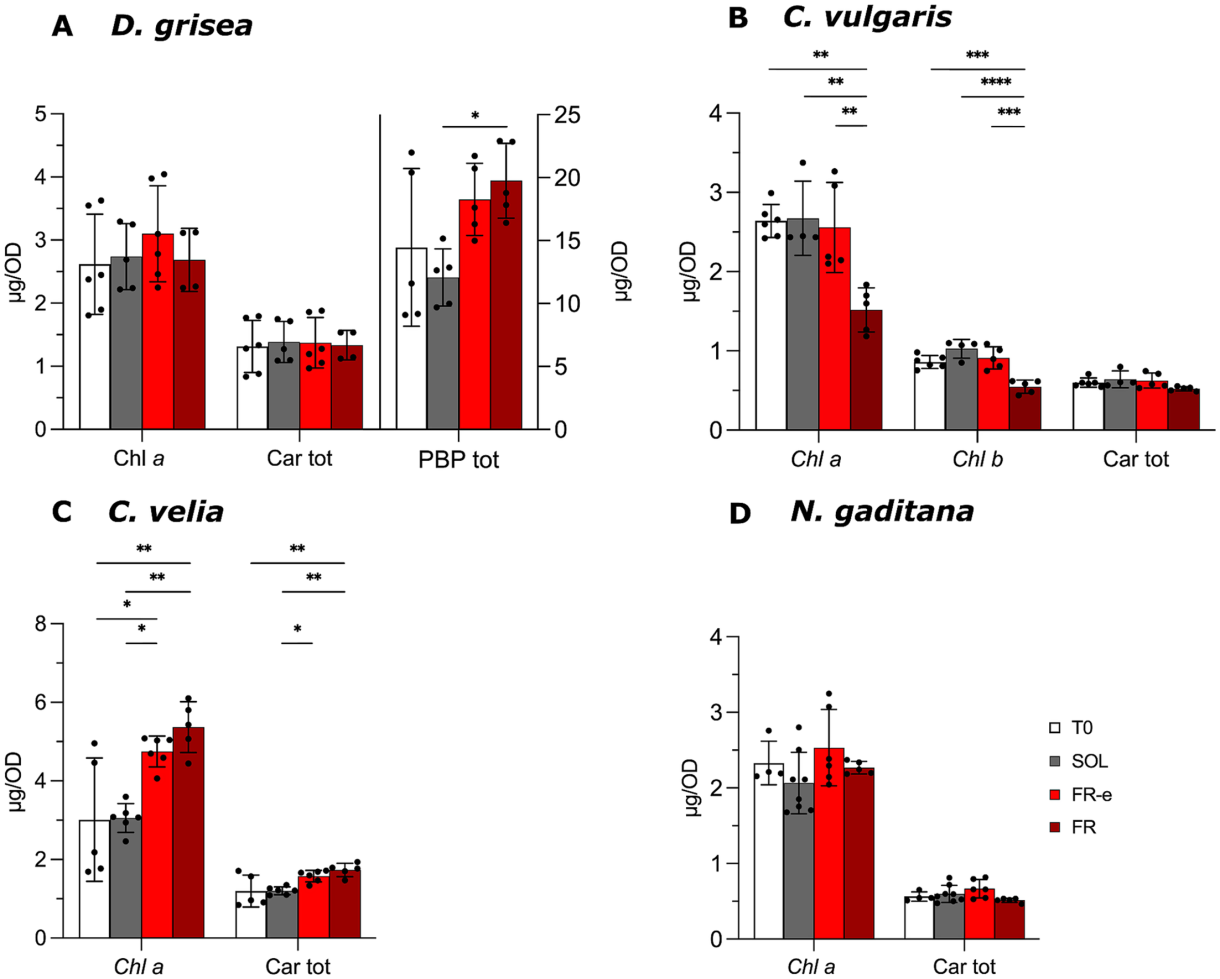
Pigment content of cells in terms of μg normalized on OD_750_ measure for each microalgal culture [*D. grisea* **(A)**, *C. vulgaris* **(B)**, *C. velia* **(C)** and *N. gaditana* **(D)**] at the starting point (T0) and at the end of the experiment in SOL, FR-e and FR light spectra. Data are expressed as mean and standard deviation of at least 3 biological replicates. Statistical analysis: Ordinary One-Way ANOVA followed by Tukey’s HSD *post-hoc* test (multiple comparisons) or Welch’s ANOVA followed by the Games–Howell *post-hoc* test depending on the dataset. Significance levels: *, *p* < 0.05; **, *p* < 0.01; ***, *p* < 0.001; ****, *p* < 0.0001. Non-significant differences are not shown. Chl *a*, Chlorophyll *a*; Chl *b*, Chlorophyll *b*; Car tot, Total Carotenoids; PBP, Phycobiliproteins.

In *D. grisea*, the total content of PBP per OD increased together with the percentage of FR photons in the light spectra, reaching the statistically significant maximum value under FR light (Figure 6A). A more detailed quantitative analysis of the content of each PBPs per OD is reported in Supplementary Figure 2. Chl *a* to total PBPs ratios (Table 2) corroborated the described results, with similar higher values for FR-e and FR with respect to SOL, indicating an increasing proportion of PBP in low VIS light.

**Table 2:** Pigment ratios calculated for each light condition (SOL, FR-e and FR).

Light spectrum	*D. grisea*		*C. vulgaris*		*C. velia*	*N. gaditana*
	Chl a/car	Chl a/PBPs	Chls/car	Chl a/Chl b	Chl a/car	Chl a/car
SOL	1.99 ± 0.10^b^	0.24 ± 0.09^a^	5.78 ± 0.12^a^	2.61 ± 0.42^a^	2.53 ± 0.15^b^	3.45 ± 0.15^c^
FR-e	2.28 ± 0.12^a^	0.18 ± 0.07^a^	5.51 ± 0.36^a^	2.79 ± 0.26^a^	3.01 ± 0.06^a^	3.87 ± 0.22^b^
FR	2.01 ± 0.03^b^	0.14 ± 0.04^a^	3.96 ± 0.66^b^	2.76 ± 0.17^a^	3.09 ± 0.11^a^	4.45 ± 0.20^a^

In particular: Chl *a* to carotenoids ratios of *D. grisea*, *C. vulgaris*, *C. velia* and *N. gaditana;* Chl *a* to PBP ratios of *D. grisea* and Chl *a* to Chl *b* ratios of *C. vulgaris.* Different letters indicate significant differences among treatments (*p* < 0.05). Statistical analysis: Ordinary One-Way ANOVA followed by Tukey’s HSD *post-hoc* test (multiple comparisons) or Welch’s ANOVA followed by the Games–Howell *post-hoc* test depending on the dataset. Chl *a*, Chlorophyll *a*; Chl *b*, Chlorophyll *b*; Car, Carotenoids; PBPs, Phycobiliproteins.

In *C. velia,* significant differences in Chl *a* content per mL were observed after 10 days across all conditions, although these variations did not fully correspond with growth data in Figure 2C. These discrepancies arose from the drop of Chl *a* and total carotenoids content per OD in SOL light at T10 with respect to FR-e and FR conditions (Figure 6C). This explains the significant decrease in Chl *a* to carotenoid ratio under this light condition (Table 2).

In *N. gaditana*, the carotenoid amount reached a higher value in SOL light than in FR-e at the end of the experiment. The Chl *a* to Car ratio (Table 2) highlights the rise of carotenoids with increased percentage of FR photons in the spectra, reaching the maximum value under FR light. Concurrently, values of Chl *a* and carotenoids per OD remain stable (Figure 6D).

Finally, in *C. vulgaris*, Chl *a* to Chl *b* ratios at T10 (Table 2) were stable in the all the light conditions. These two pigments underwent a significant decrease under monochromatic FR light, while the carotenoid level remained the same. This reflects in a change in Chls to Car ratio, that is significantly lower at T10 under FR light than the two other conditions.

To characterize pigment profiles, qualitative HPLC analyses were performed on T10 for each light treatment and microalgal culture. Pigment identity and relative elution order for each species were consistent with literature (Alboresi et al., 2016; Basso et al., 2014; Jeffrey et al., 1997; Moore et al., 2008; Quigg et al., 2012; Serive et al., 2017; Takacs et al., 2025; Wright et al., 1991) and did not change in composition across all tested conditions (Supplementary Figure 3 and Supplementary Table 3).

## 4 Discussion

A limiting factor for the biomass productivity in BLSS, as well as biotechnology systems, is the high cellular density in photobioreactors (Fahrion et al., 2021) which leads to a progressive internal enrichment in FR wavelengths, due to the preferential absorption of VIS light by the outer cell layers (Saccardo et al., 2022). In this work we analyzed how different species of microalgae, capable or incapable of utilizing FR light for photosynthesis, respond to alternative light sources differently enriched in FR wavelengths.

As non-FR users we selected *D. grisea* and *C. vulgaris*, originating from primary endosymbiosis and representing the red and green lineages of the Archaeplastida, respectively.

As regards the FR-users, we focused on microalgae originating from secondary endosymbiosis and belonging to the SAR (Stramenopiles, Alveolates, Rhizaria) supergroup, in which FR-driven photosynthesis is more widespread. The selection was based on the principal adaptive strategies for Far-Red (FR) light utilization described to date. The first involves a red shift of Chl *a* absorption resulting from rearrangements of the surrounding protein environment within pigment–protein complexes. This acclimation was observed in the green alga *Ostreobium* (e.g., *O.* sp., *O. quekettii*), and between SAR, in the chromerid *C. veli*a, the eustigmatophyte FP5 and *Trachydiscus minutus*, and the diatoms *Phaeodactylum tricornutum* and *Nitzschia closterium* (Fujita and Ohki, 2004; Litvín et al., 2016; Pazderník, 2015; Wilhelm and Jakob, 2006; Wolf and Blankenship, 2019).

Interestingly, recent studies demonstrated that an increased absorption shoulder beyond 700 nm is not universally detected in microalgae capable of FR light utilization (Chen et al., 2025; Liistro et al., 2026). In this regard, an in-depth investigation recently carried out in our laboratory on the eustigmatophyte *N. gaditana*, described a new strategy that relies on the constitutive capacity of photosystem I (PS I) to harvest limited amount of FR photons without requiring a specific acclimation (Liistro et al., 2026). This ability resulted also coupled with the ultrastructural remodeling of the photosynthetic apparatus, building organized thylakoidal bodies of membranes, which seem to enhance the low-energy FR light use (Liistro et al., 2026). Such a strategy in *N. gaditana* leads to an unchanged *in vivo* absorption spectrum after FR acclimation compared to SOL light (Liistro et al., 2026).

In light of this information, for the present work we selected *N. gaditana* and *C. velia* to analyze their responses to a FR-e spectrum, as representatives of the two distinct FR acclimation strategies: the Chl *a* red shift in *C. velia* (Kotabová et al., 2014) and the constitutive PSI FR harvesting associated to the chloroplast membrane remodeling in *N. gaditana* (Liistro et al., 2026).

First, the growth capacity and the acclimation responses were analyzed for each selected species.

Growth tests yielded robust results under FR-e light compared with SOL. The increase in OD over 10 days of experiment was considerable in all tested species (Figure 2). Among the FR-utilizing species*, C. velia* and *N. gaditana* showed particularly high OD increments under FR-e. In *N. gaditana*, OD increased 3.3-fold in SOL and 2.9-fold in FR-e. In *C. velia*, OD increased by approximately 2-fold under both light conditions. Among the species unable to utilize FR, the increments were slightly lower but still substantial. In *D. grisea*, OD increased 1.4-fold in SOL and 0.9-fold in FR-e, while in *C. vulgaris*, OD increased 3.7-fold in SOL and approximately 1.9-fold in FR-e.

Considering that FR-e light contains only 37% of photons in the VIS, 400 and 700 nm, whereas in the SOL spectrum nearly the entire photon flux is concentrated in this range (84.8%) (Figure 1; Supplementary Table 1), these OD increases at the end of the experiment are markedly higher than expected based on the percentage of VIS photons in the applied spectra.

To fully understand the importance of these results, growth under FR must also be carefully considered. In the non-FR-utilizing species, the OD increment under FR was approximately 0% at T10 (Figure 2). Even if *C. vulgaris* appeared to grow under FR, the color of the cultures and their pigment content (Supplementary Figure 1) did not reflect the measured OD low increase over 10 days. This increment was therefore mainly attributable to light scattering by dead cells. Among the FR-users, *C. velia* exhibited the highest growth under FR over 10 days, reaching a 150% increase relative to the initial OD, whereas *N. gaditana* showed a 77% increase relative to the initial OD. This growth difference may be explained by the different acclimation strategies: *N. gaditana* constitutively absorbs a small fraction of FR light via PSI, whereas *C. velia* undergoes an active shift in Chl *a* absorption properties, that seems to maximize the capture of incident FR light. The capacity of *C. velia* of harvesting the FR light finds good evidence in the *in vivo* spectroscopy (Figure 5C), where a FR dose-dependent acclimation occurs generating an absorption shoulder above 700 nm, more pronounced under monochromatic 730 nm FR light and less in FR-e. A similar pattern was observed in the cyanobacterium *Chlorogloeopsis fritschii* during FaRLiP acclimation (Far-Red Light Photoacclimation), where the absorption shoulder above 700 nm increased with FR photon proportion between FR-e, and FR under comparable total irradiance (Battistuzzi et al., 2023a). It is interesting to note the analogous FR dose-dependent spectral signature in *C. velia* respect to *C. fritschii,* despite the different underlying FR acclimation mechanisms. In the cyanobacterium the response is in fact driven by the synthesis of Chl *d*, Chl *f*, and FR allophycocyanin (Battistuzzi et al., 2024; Ho and Bryant, 2019). Contrarily, the cyanobacterium *Synechococcus* sp. PCC 7335, capable of both type 3 chromatic acclimation (CCA3) and FaRLiP, activates CCA3 under FR-e, adjusting PBP ratios rather than synthesizing Chls *d* and *f* (Liistro et al., 2024).

Overall, our results, show a good capacity of all tested microalgae to grow under the FR-e spectrum with respect to SOL, namely 61% in *C. vulgaris*, 82% in *D. grisea*, 91% in *N. gaditana*, and 93% in *C. velia*. This cannot be explained just considering the FR use efficiency of the FR-user strains but need to consider the beneficial Emerson effect possible for all species (Emerson et al., 1957). The Emerson enhancement effect states that the photosynthetic efficiency increases when long-wavelength photons (680–720 nm) are combined with shorter-wavelength photons (Zhen et al., 2022). This phenomenon can justify also the considerable growth under the FR-e spectrum of *D. grisea* and *C. vulgaris*, non-FR-users. Yet the much higher growth performances of *C. velia* and *N. gaditana* under the same light spectrum can be explained by the FR-driven photosynthesis, which adds to the Emerson effect.

A second important observation is that the biomasses obtained under the different light conditions for each microalgal strain did not show significant changes in cell features (Figure 4) and their specific pigment composition as the HPLC analysis reveals (Supplementary Figure 3).

Moreover, in contrast to previous reports on *Dunaliella bardawil* exposed to WL supplemented with FR light (Sánchez-Saavedra et al., 1996), no high carotenoid accumulation was detected in the present study in any microalgal species under SOL, including the FR low-energy band, as well as under the low-VIS FR-e spectrum. Total chlorophylls, carotenoids and phycobiliproteins per OD were mostly stable.

Of interest, in *D. grisea*, the high-value-pigments PBPs tended to increase their mean value per OD (Figure 6A; Supplementary Figure 2) and their ratio relative to Chl *a* (Table 2) with higher FR photon proportion.

In *C. velia*, the only differences observed occurred under SOL light. A reduction in pigment content per OD was observed (Figure 6C), reflected in the decrease of the Chl a/Car which highlights a Chl *a* reduction more marked than Car (Table 2). This data suggests a possible mild stress responses which likely occur in the higher VIS photon flux, consistent with the natural ecology of this species as a coral symbiont adapted to low-light environments (Wangpraseurt et al., 2012).

Finally, in *N. gaditana* light absorption resulted comparable under FR-e and FR conditions, whereas under SOL differs, with higher absorption in the carotenoid range (Figure 4). The Chl *a*/Car ratios reveal significant differences (Table 2) with an increase under FR-e and highest values under FR. These results agree with previous studies on *N. gaditana* grown under FR light, where Chl *a* markedly increased with respect to SOL light (Liistro et al., 2026). Moreover, it is interesting to note that in *N. gaditana* the increase of thylakoidal membranes under FR light (Liistro et al., 2026), may in turn promote the production of the membrane omega-3 fatty acids.

Overall, our results allowed us to expand the current knowledge on microalgal responses to FR light (still poorly understood) and to investigate how these photosynthetic microorganisms respond to low VIS light enriched in FR wavelengths. This light regime turned out to be effective for all tested species, enabling high biomass production without exhibiting major or negative alterations of cell morphology and pigment composition. Moreover, considering that biomass increases largely exceed the proportional differences in high-energy photon availability between the tested spectra, at least the faster growing species here investigated could represent promising candidates for high-density cultivation in closed systems such as photobioreactors used in space biotechnology.

However, calculations of the total energy (expressed in W m^−2^) of the FR-e spectrum (83.5% of SOL) indicate that this light regime is particularly advantageous for the cultivation of FR-user *N. gaditana* which achieved 91% of the growth observed under SOL. This percentage is much higher than that registered for *C. vulgaris* (61%). These relevant findings identify *N. gaditana* as a promising candidate for BLSS application, due to its high growth rate and the capability to use FR light beside the VIS one.

In this context, it will also be important to investigate how biomass productivity and the synthesis of bioactive compounds, such as omega-3 fatty acids, are affected by this light regime, coupled with the cultivation using astronaut waste (e.g., urea and CO₂) and lunar and Martian soil simulants (Casula et al., 2024; Niederwieser et al., 2018).

Semicontinuous and continuous long-term growth under FR-e and other optimized parameters such as temperature, nutrient availability as well as the photobioreactor geometry and capacity, could help to optimize the biomass and high-value molecule production for BLSS purposes (Detrell, 2021; Revellame et al., 2024; Verseux et al., 2022).

Energy and cooling costs are widely variable depending on the bioreactor arrangement and environmental conditions. In our case, only passive cooling was needed to operate our simulators at the required power levels. However, as a general consideration, radiometric efficiency of current state of the art LEDs shows a drop in the green region known as the “green gap,” either for light obtained through direct emission LEDs (due to material limitations and reduced internal quantum efficiency) and for white phosphor LEDs (due to conversion losses). Blue LEDs as well as Far-Red devices show the highest efficiencies; for those reasons, the Far-Red spectral region is particularly appealing in lighting for highly energy-efficient photosynthesis.

Moreover, reducing light input provides a dual benefit: it decreases the energy demand for lighting while simultaneously lowering the associated cooling requirements, which may constitute a significant fraction of overall system cost. Indeed, passive cooling options are severely limited in space systems, and space-based photobioreactors require more extensive active thermal control to dissipate excess heat than comparable terrestrial systems. These considerations further underscore the significance of the results presented in this study.

With the roadmap of upcoming long-term space missions, such as NASA’s Artemis program aimed at establishing a sustained human presence on the Moon, ESA’s Moonlight program lunar infrastructure initiative, and future crewed missions to Mars envisioned by NASA and ESA, the development of energy-efficient and autonomous BLSS is increasingly critical. Microalgae are key candidates, but their productivity and biomass quality is strongly influenced by light regimes.

The information on light requirements obtained for BLSS and biotechnological purposes can also find applications in astrobiology. Investigating how microalgae respond to FR-enriched radiation also helps constrain the spectral boundaries within which oxygenic photosynthetic habitability is allowed. Their identification is critical for evaluating whether photosynthetic life as we know it could have evolved and remained productive under alternative stellar spectra. In fact, surface radiative environments modeled for exoplanets orbiting M-dwarf stars are enriched in FR and near-infrared wavelengths, with a spectrum that closely resembles the one we simulated in this work (Pecaut and Mamajek, 2013). This category of stars is among the most common ones in our galaxy and host the majority of currently identified potentially habitable exoplanets (Shields et al., 2016). Furthermore, similar considerations apply within our own Solar System. Early Mars, subjected to intense ultraviolet radiation from the faint young Sun and lacking a protective ozone layer, may have provided more favorable conditions for life in UV shielded subsurface niches (Godin et al., 2023; Viúdez-Moreiras, 2021). In such protected environments, light spectra could resemble our FR-enriched conditions. For these reasons, exploring the physiological plasticity of microalgae and limits of oxygenic eukaryotic phototrophs under these spectra therefore informs not only BLSS optimization strategies, but also models of subsurface habitability on early Mars and exoplanets.

## Data Availability

The raw data supporting the conclusions of this article will be made available by the authors, without undue reservation.
