# Supplementary material for: Growth of phylogenetically diverse microalgae under Far-Red light enriched spectra: implication for space missions’ sustainability
Source: Front Microbiol. 2026 May 19;17:1833797. doi: 10.3389/fmicb.2026.1833797 (PMC13226475; doi:10.3389/fmicb.2026.1833797)
Supplement: Supplementary file 1 [file Data_Sheet_1.pdf]

Supplementary Material

1 Supplementary Figures and Tables

Supplementary Table 1. – Photon flux specification of SOL, FR-e and FR spectra reproduced by the simulators and revealed by the LI-COR 180 spectrometer (LICOR) in the spectral range (380-780 nm). PPFD, Photosynthetic Photon flux density; PFD, Photon flux density; PFD-FR, Photon flux density in the Far-Red wavelength range. These measurements are indicated in µmol photons m^{-2} s^{-1}. UV%, percentage of photons in the UV range; VIS%, percentage of photons in the visible range; FR%, percentage of photons in the Far-Red range; B%, G% and R%, percentage of photons respectively in blue, green and red wavelength range. W/m^2, energy of photons in the 380-780 nm spectral range.

	Spectral range	SOL	FR-e	FR
PPFD	**400-700 nm**	25.3	11.1	22.8
PFD-FR	**700-780 nm**	45.6	18.8	27.9
PFD	**380-780 nm**	30.0	30.0	30.2
UV%	**380-400 nm**	0.50	0.20	0.04
VIS%	**400-700 nm**	84.3	37.0	7.56
FR%	**700-780 nm**	15.2	62.8	92.4
B%	**400-500 nm**	23.2	4.40	0.2
G%	**500-600 nm**	29.8	12.2	0.2
R%	**600-700 nm**	31.4	20.4	7.14
$W\ m^{-2}$	**380-780 nm**	6.30	5.26	4.83

Supplementary Table 2. – Maximal average growth rates μ_{max} (d^{-1}) of *D. grisea*, *C. vulgaris*, *C. velia* and *N. gaditana* cultures exposed to the different light spectra (SOL, FR-e and FR). The letters *a* and *b* indicate significant differences among treatments ($P < 0.05$). Statistical analysis: Ordinary One-Way ANOVA followed by Tukey's HSD post-hoc test (multiple comparisons) or Welch's ANOVA followed by the Games–Howell post hoc test depending on the dataset.

	**Maximal growth rate**		
**Species**	**SOL**	**FR-e**	**FR**
D. grisea	0.138 ± 0.044^{a}	0.116 ± 0.063^{a}	$0{,}029 \pm 0{,}010^{b}$
C. vulgaris	0.197 ± 0.070^{a}	0.134 ± 0.035^{a}	0.028 ± 0.022^{b}
C. velia	0.156 ± 0.028^{a}	0.140 ± 0.043^{a}	0.123 ± 0.053^{a}
N. gaditana	0.343 ± 0.145^{a}	0.278 ± 0.140^{b}	0.087 ± 0.154^{c}

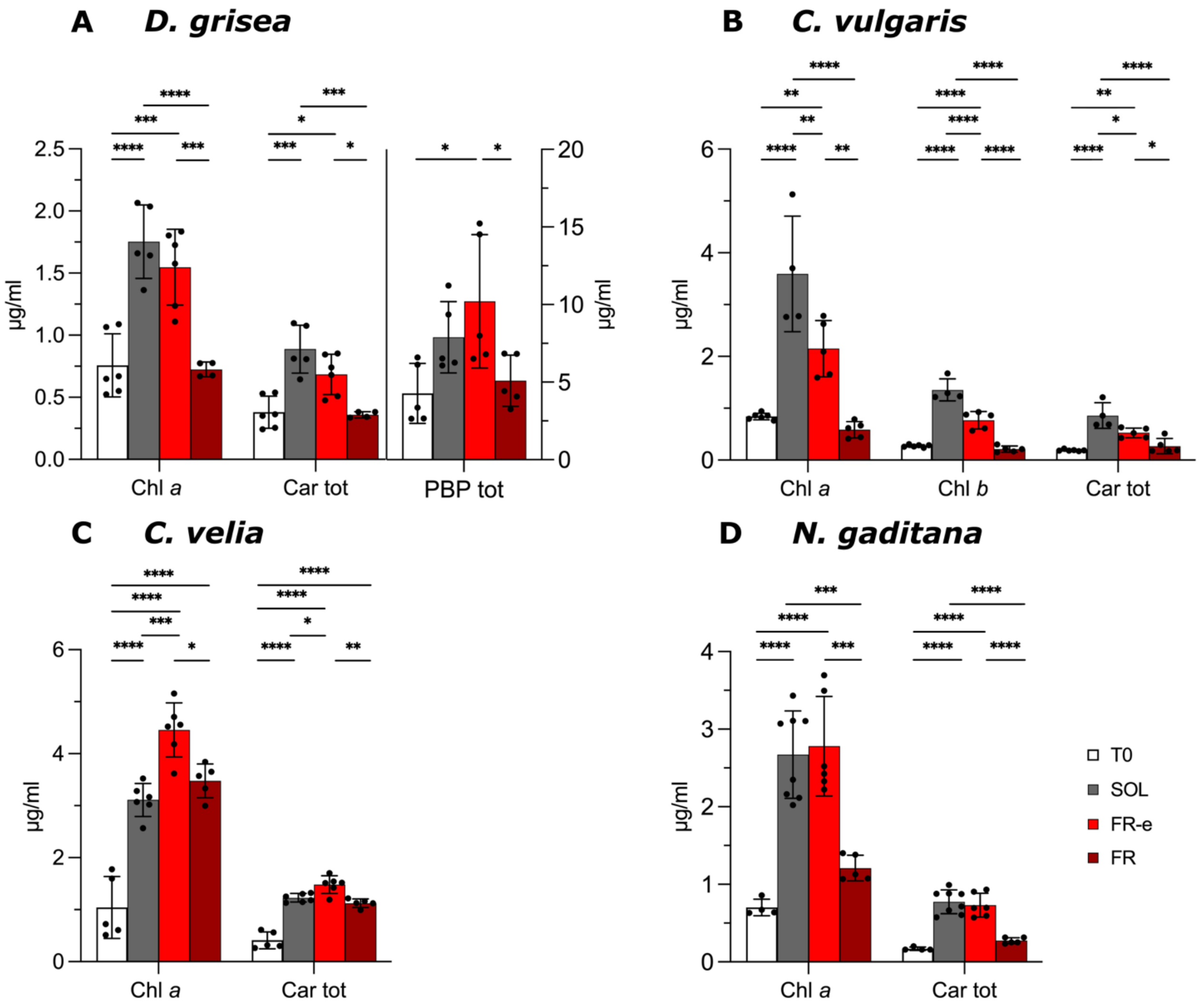

Supplementary Figure 1. Pigment content of cells in terms of µg of pigments per ml of microalgal culture of respectively *D. grisea* (A), *C. vulgaris* (B), *C. velia* (C) and *N. gaditana* (D) at the starting point (T0) and at the end of the experiment (T10) in SOL, FR-e and FR light spectra. Data are expressed as mean and standard deviation of at least 3 biological replicates. Statistical analysis: Ordinary One-Way ANOVA followed by Tukey's HSD post-hoc test (multiple comparisons) or Welch's ANOVA followed by the Games–Howell post hoc test depending on the dataset. Significance levels: *, $P < 0.05$; **, $P < 0.01$; ***, $P < 0.001$; ****, $P < 0.0001$. Non-significant differences are not shown. Chl *a*: Chlorophyll *a*; Chl *b*: Chlorophyll *b*; Car tot: Total Carotenoids; PBP: Phycobiliproteins.

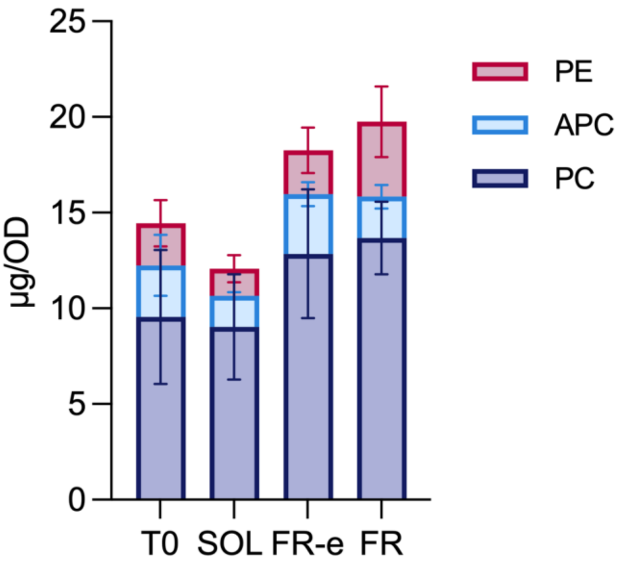

Supplementary Figure 2. Quantification of phycobiliproteins in *Dixoniella grisea* at the starting point (T0) and at the end of the experiment (T10) in SOL, FR-e and FR light spectra. PE: Phycoerythrin; APC: Allophycocyanin; PC: Phycocyanin. Data are expressed as mean and standard deviation of at least 3 biological replicates. Statistical analysis: Ordinary One-Way ANOVA followed by Tukey's HSD post-hoc test (multiple comparisons) or Welch's ANOVA followed by the Games–Howell post hoc test depending on the dataset. There is no statistically significant difference between the reported data.

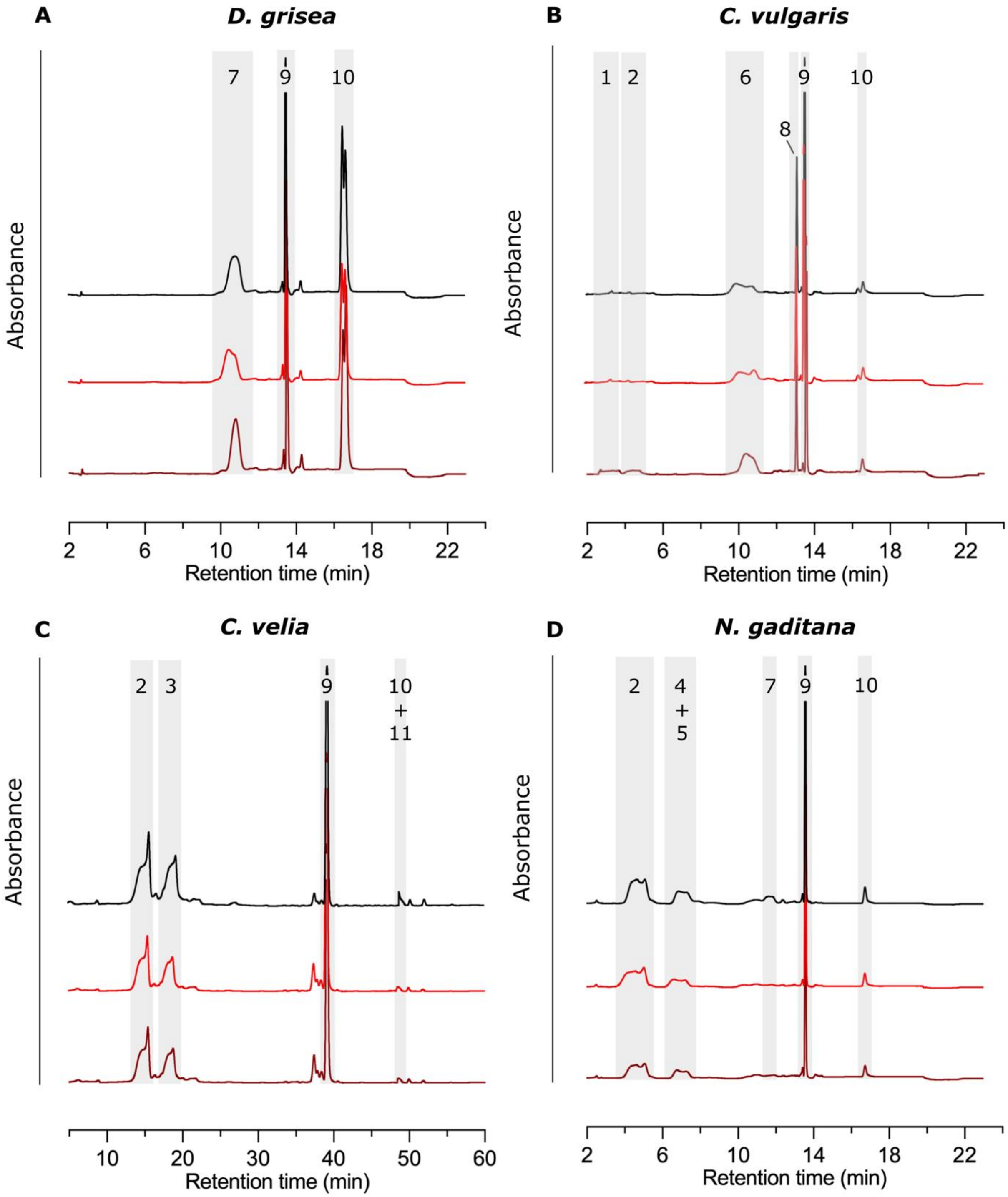

Supplementary Figure 3. HPLC chromatograms of *D. grisea*, *C. vulgaris*, *C. velia* and *N. gaditana* at the final day of the experiment (T10) in SOL, FR-e and FR. The chromatograms are normalized at the absorbance of Chl *a.* The identified compounds are reported in Supplementary Table 3 with their absorption maxima (λ max).

Supplementary Table 3. – Specification of HPLC analysis in Supplementary Figure 3. Pigments are indicated in order of elution. The maximum peaks of each spectrum are indicated in nm. Blank cells indicate the absence of the peak. For the first peak, where present, an approximate value is indicated due to its high variability.

Number	Pigment	λ max in eluant (nm)		
**1**	**Neoxanthin**	~ 414	434	466
**2**	**Violaxanthin**	~ 418	440	470
**3**	**Chromeraxanthin**			470
**4**	**Vaucheriaxanthin**	~ 418	444	472
**5**	**Antheraxanthin**	~ 418	445	474
**6**	**Lutein**	~ 422	446	474
**7**	**Zeaxanthin**	~ 420	454	480
**8**	**Chl *b***		466	650
**9**	**Chl *a***		432	664
**10**	**β-carotene**	~ 422	452	478
**11**	**Cis-β-carotene**	~ 423	446	470
